# In Search of a Green Process: Polymeric Films with Ordered Arrays via a Water Droplet Technique

**DOI:** 10.3390/polym11091473

**Published:** 2019-09-09

**Authors:** Shih-Chieh Yeh, Chien-Hsin Wu, Ying-Chih Huang, Jen-Yu Lee, Ru-Jong Jeng

**Affiliations:** 1 Institute of Polymer Science and Engineering, National Taiwan University, Taipei 106, Taiwan; 2 Advanced Research Center for Green Materials Science and Technology, National Taiwan University, Taipei 106, Taiwan

**Keywords:** breath figure (BF) method, ordered arrays, water droplets, green process

## Abstract

As an efficient technique for the preparation of polymeric hexagonal orderly arrays, the breath figure (BF) process has opened a modern avenue for a bottom-up fabrication method for more than two decades. Through the use of the water vapor condensation on the solution surface, the water droplets will hexagonally pack into ordered arrays, acting as a template for controlling the regular micro patterns of polymeric films. Comparing to the top-down techniques, such as lithography or chemical etching, the use of water vapor as the template provides a simple fabrication process with sustainability. However, using highly hazardous solvents such as chloroform, carbon disulfide (CS_2_), benzene, dichloromethane, etc., to dissolve polymers might hinder the development toward green processes based on this technique. In this review, we will touch upon the contemporary techniques of the BF process, including its up-to-date applications first. More importantly, the search of greener processes along with less hazardous solvents for the possibility of a more sustainable BF process is the focal point of this review.

## 1. Introduction

**Figure 1:**
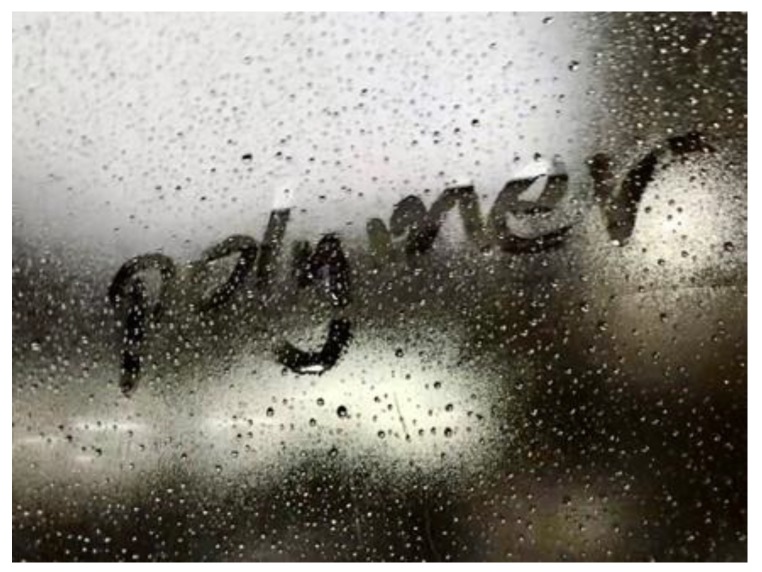
Breath fog on the window.

Breath figure (BF) is an example of the nature phenomenon of the formation of water droplets on surfaces (Figure 1). Since the first discovery in 1911 [1,2], for morphology observation, and further development by Francois et al. [3] in 1994 for materials science, the BF process has become one of the most promising fabrication methods in the production of porous polymeric films with ordered arrays. These pore structures, typically exhibiting a regular hexagonal arrangement, are usually regarded as honeycomb-like polymeric films. Unlike the traditional top-down techniques, such as lithography, etching, or the chemical vapor deposition method for regular patterns, the BF process provides a low cost, simple, and efficient route toward polymeric films with ordered arrays by the use of the self-assembly of water droplets on the surface.

**Figure 2:**
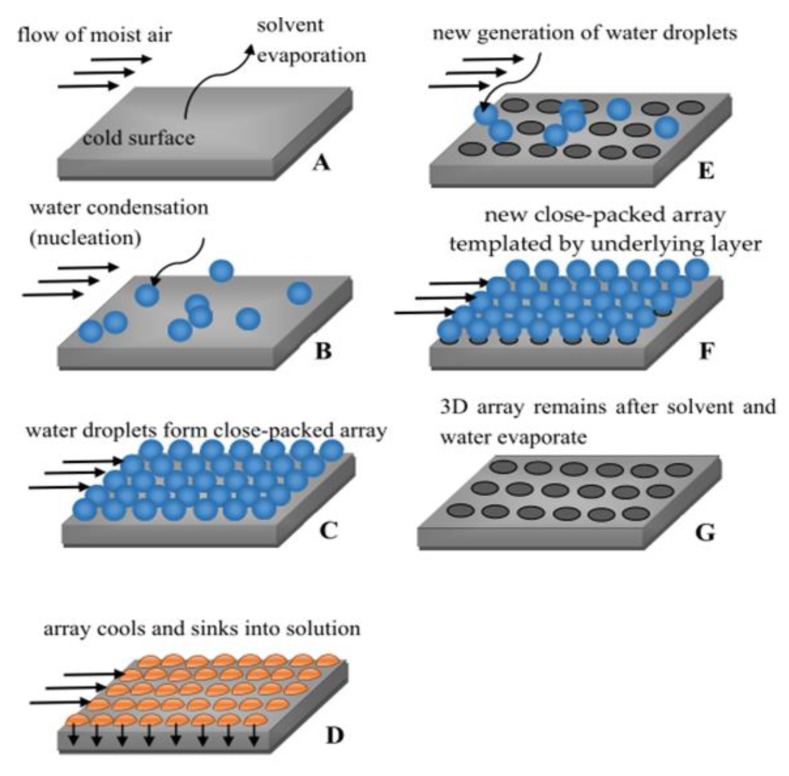
The sequence of stages during the formation of honeycomb-like films via the BF process. © reprinted from [5] under open access license.

Although the detail mechanism is profound due to the complex mass and heat transfer during the formation of ordered array films, the comprehensive illustration of the BF process is described in Figure 2. First, a polymer solution is drop-cast or solution-cast on a substrate (step A). Subsequently, the cooling of the solution and the nucleation of the moisture occur simultaneously, producing small but disordered water droplets on the solution surface (step B). As time goes by, the self-assembly of the water droplets would form an ordered and closely-packed water droplet array that covers the entire surface of the solution (step C and D), followed by the evaporation of the solvent and water droplets, leaving a regular pore array on the dry film (step E, F, G). Due to certain parameters being controllable from B to G steps, specific ordered arrays could be handled by changing the types of materials, substrates, solvents, and the size of water droplets. For a further detailed description of the BF process one can refer to the literature reported by Dou et al. and Bormashenko [4,5].

In most cases, the water vapor is utilized as templates floating on the solution to fabricate the polymeric honeycomb-like films. As a result, the BF process is a promising candidate for a green process that fabricates micropatterns with good sustainability. However, the use of highly hazardous solvents such as chloroform, carbon disulfide (CS_2_), dichloromethane, or benzene for dissolving polymers would stand in the way of the development of a greener BF process. In order to reduce the environmental impact, green chemical processes and synthesis design are strenuously developed for improving chemical ingredient manufacturing, nanotechnology, flow chemistry, and process intensification under harsh synthesis conditions [6]. In one example, a record high-power conversion efficiency for rapidly-developed organic photovoltaics (OPVs) was boosted up to 16% [7]. However, the active layers of state-of-the-art OPVs were processed by highly hazardous chlorinated solvents, such as 1,2-dichlorobenzene (DCB), chlorobenzene (CB), and chloroform (CF), which put public health and the environment in danger. With great endeavor in the pursuit of greener process, OPVs prepared from o-xylene could be achieved up to 13% more recently [8]. Based on the above, the solvent issue plays a highly critical role in the fabrication of polymeric thin films. With the right choice of solvents, the facile BF process, via water droplets as a template, can be regarded as a green and safe process for fabricating polymeric ordered arrays.

In this review, recently-developed materials for polymeric honeycomb-like films will be elucidated first, followed by the applications of these polymeric honeycomb-like films. Subsequently, we will discuss the feasibility of greener processes for fabricating the polymeric honeycomb-like films. The maneuverable BF process does hold the opportunity for a more sustainable process without using highly hazardous solvents. Finally, a summary and an outlook on this greener BF process are presented at the end of this review.

## 2. Materials

In the past two decades, a large number of polymers have been developed for fabricating honeycomb-like films with micro- and nano-structures by a dynamic templating technique, namely the BF process [4,5,9,10,11,12,13,14,15,16]. Some features such as (1) high quality of film forming; (2) water immiscible, and (3) efficient stabilization of water droplets are considered to afford a polymer solution for preparing honeycomb-like films by the BF method. Among these polymers, amphiphilic polymers [16,17,18,19] and functionalized block copolymers [20,21,22], capable of balancing the hydrophobicity and hydrophilicity of the solution–water interface, are favorable for forming ordered arrays during the BF process. In one particular example, an ionic group/counter-ion effect on porous polymer film morphology was investigated based on a series of PH3T-b-PMMA diblock copolymers [20]. In addition, a series of amphiphilic hyperbranched polymers were developed by Dong et al. to obtain 5–6 μm diameter pores, and the depth of pores were increased with increasing solution concentration [23]. Apart from that, star and branch polymers with high segment density were also one of the popular materials for fabricating honeycomb-like films [24,25,26,27,28,29]. On the other hand, certain small molecules with special functional groups, such as melamine units, would induce a supramolecular assembly through the interactions of hydrogen bonding. This makes it possible to form ordered honeycomb-like films via the BF method [30]. It is important to note that surfactants were capable of acting as the driving force to induce the self-assemble behavior in the formation of ordered arrays [31,32,33]. Furthermore, honeycomb-like films could be fabricated based on certain polymers used as matrices for nanoparticles (NPs), carbon nanotubes (CNTs), quantum-dot [34], or graphenes [35]. In one example, ordered arrays of an elastomer–CNT nanocomposite were achieved based on the mixture of styrene–butadiene–styrene (SBS) and amine-terminated polystyrene (PS-NH2) solution [36]. Apart from that, the self-assembly of nanoparticles at the oil–water interface (Pickering emulsions) in the BF process have been widely utilized for honeycomb-like micropatterning [35,37,38,39,40,41,42]. In fact, rings of nanoparticle-decorated honeycomb-like polymeric films could be observed with the combination of Pickering emulsions and capillary flow via the BF method [40]. Researchers also fabricated honeycomb-like films built on a photo-curable and biodegradable polycaprolactone triacrylate (PCLTA) [43]. UV light was irradiated onto the sample at the stage of water droplet condensation. Subsequently, the ordered arrays were formed when tetrahydrofuran (THF) was evaporated completely.

### 2.1. Amphiphilic Polymers for Honeycomb-Like Films

The effects of hydrophobicity and hydrophilicity could be manipulated by the variations of the polymer end groups. Zhu et al. [44] synthesized several polystyrenes (PSs) with different functional end groups, and fabricated honeycomb-like films from their polymer solutions (in CS_2_) via the BF process. Highly-ordered honeycomb-like films were obtained from the PSs with ionized or neutralized end groups, whereas the irregular structure was observed on the PS sample with a less hydrophilic lactone end group. These results reveal that various morphologies of honeycomb-like films could be achieved by these well-controlled end-functionalized polymers.

**Figure 3:**
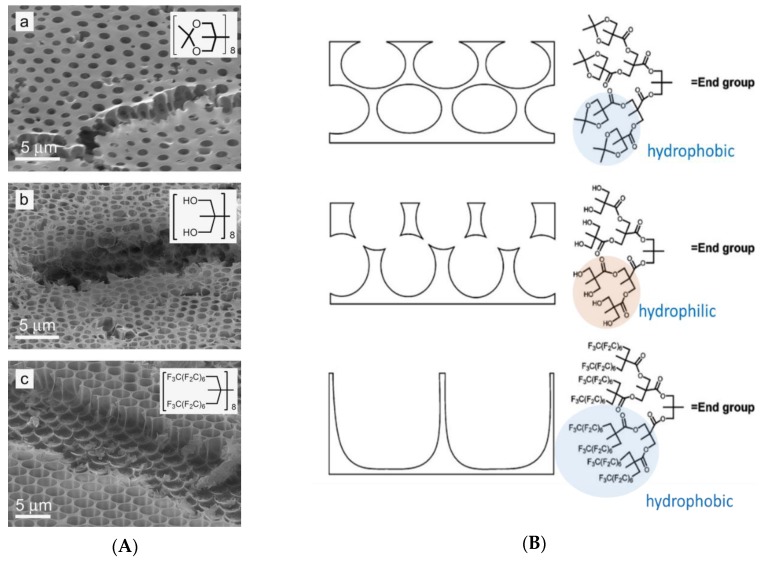
Honeycomb-like films fabricated with various end groups based on bis-MPA dendrons: (**A**) SEM micrographs of the honeycomb-like film made from star-shaped dendron-functionalized with a. acetonide-functionalized G3 star polymer; b. hydroxyl-functionalized G3 star polymer; c. perfluoroalkyl-functionalized G3 star polymer. Insets show end-group structure; (**B**) schematic representation of honeycomb films change with the end groups [45]. © Reproduced with permission from Wiley.

Connal et al. [45] reported the preparation of an acetonide-protected dendron-functionalized star polymer (polystyrene based) for fabricating honeycomb-like films. The dendrons were end-functionalized with acetonide-protected (hydrophobic), hydroxyl (hydrophilic), or perfluoroalkyl (highly hydrophobic) groups. Various pore sizes and shapes of honeycomb-like films could be facilely achieved based on the polystyrene-based star polymers end-functionalized with 2,2-bis(methoxy)propionic acid-based dendrons using benzene as the solvent via the BF process (Figure 3).

**Figure 4:**
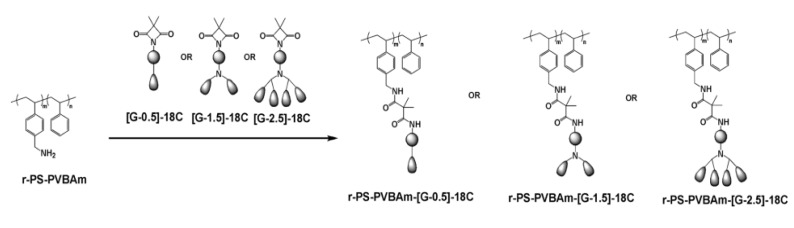
Polystyrene with grafting of [G-0.5], [G-1.5], and [G-2.5] dendrons [27]. © Reproduced with permission from the Royal Society of Chemistry (RSC).

**Figure 5:**
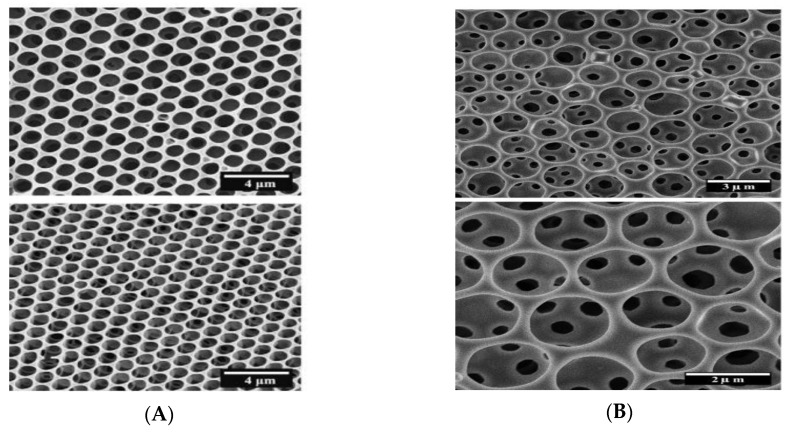
SEM images of PS dendritic polymers honeycomb-like films on Si wafer for polymer solution (in chloroform): (**A**) PU-[G2.5]-18C-diol-HDI films [25]; (**B**) *r*-PS-PVBAm-*g*-[G-1.5]-18C [27]. © reprinted from [25] with Elsevier permission of Elsevier; © Reproduced from [27] the with permission of Royal Society of Chemistry (RSC).

Amphiphilic poly(urea/malonamide) dendritic materials have been developed by Jeng et al. since 2006 [25,26,27,28,29,46,47,48,49,50,51,52,53,54,55,56,57,58,59,60,61]. In the midst of them, the honeycomb-like films were obtained based on PSs covalently bonded with different sizes of dendritic side chains (in chloroform) (Figure 4). The presence of these dendritic side chains comprising long alkyl chains in the periphery and hydrogen bond-rich urea/malonamide linkages in the focal part helps induce self-assembly and phase-separation in the formation of honeycomb-like polymeric films by the BF method (Figure 5) [25,27].

### 2.2. Surfactant-Facilitated BF Process for Honeycomb-Like Films

**Figure 6:**
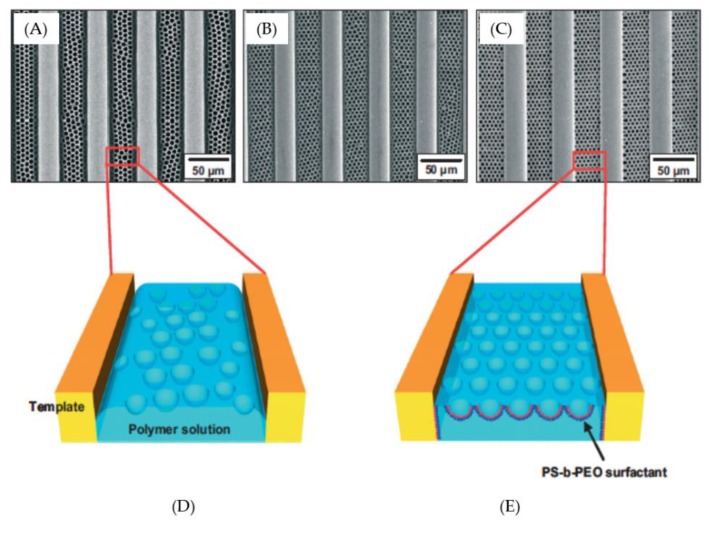
SEM images of various polymeric surfactants of polystyrene-block-poly(ethylene oxide) contains, in a 4.0 wt% polystyrene polymer solution: (**A**) 0 wt%; (**B**) 0.4 wt%; (**C**) 0.8 wt%. (**D**,**E**) Schematic comparisons of the (**D**) poor wetting between the polymer solution and grating surface, and (**E**) the red chain indicates the addition of hydrophilic PEO blocks and the blue chain indicates the hydrophobic PS blocks [31]. © Reproduced with permission from Wiley.

Park et al. [31] attempted to fabricate a hierarchically-ordered polymeric film by templating the organization of aqueous droplets. In the study, a highly-ordered structure that can be tuned by dissolving a small amount of surfactant (polystyrene-block-poly(ethylene oxide) in the polymer solution (PS in benzene) (Figure 6). This lithography-free fabrication method provides a new opportunity for the complex hierarchical structures. Recently, Zhang et al. [33] developed a magnetic honeycomb-like structure on the indium tin oxide substrate for electrocatalysis based on a surfactant-encapsulated polyoxometalate complex, in which dimethyldioctadecylammonium bromide acted as the surfactant in a chloroform solution and would induce self-organization honeycomb-like patterns.

**Figure 7:**
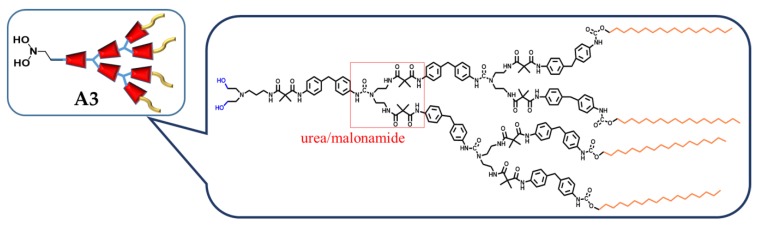
Schematic drawing of the dendritic poly(urea/malonamide) surfactant (A3). © Reproduced with permission from Royal Society of Chemistry (RSC).

**Figure 8:**
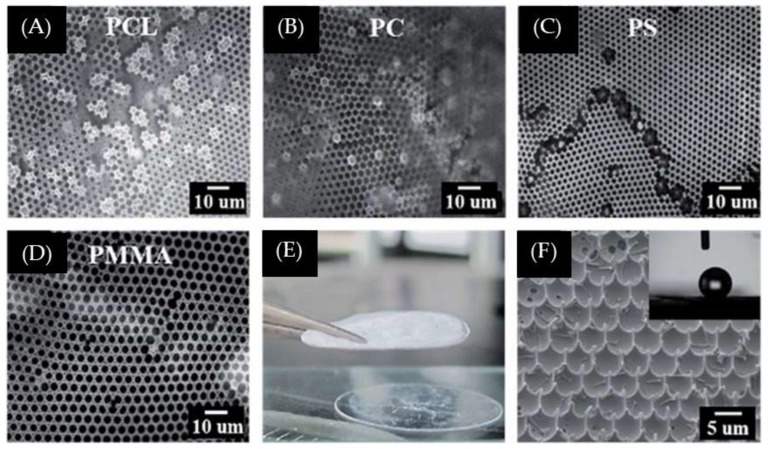
Images of honeycomb-like surfaces obtained from the BF process (95% RH) based on a polymer solution comprising dendron (A3) in chloroform (10 mg/mL) [28]: (**A**) A3/PS; (**B**) A3/PCL; (**C**) A3/PC; and (**D**) A3/PMMA from microscope; (**E**) photograph of free-standing A3/PMMA film; and (**F**) image of the superhydrophobic surface of the A3/PS honeycomb-like film after the peeling-off process. © Reproduced with permission from the Royal Society of Chemistry (RSC).

Amphiphilic poly(urea/malonamide) dendrons developed by Wu et al. [28] were also utilized as surfactants to facilitate the formation of honeycomb-like porous structures from the BF process (Figure 7). These dendrons are amphiphilic, with a hydrogen-bond-rich focal part and a periphery with nonpolar units that undergo van der Waals interactions. With the addition of a small amount of dendritic surfactants to the polymer solutions, such as poly(d,l-lactide), PS, poly(methyl methacrylate) (PMMA), or polycarbonate (PC) in chloroform, a free-standing film with a honeycomb-like surface could be achieved (Figure 8).

### 2.3. Crosslinkable Materials for Honeycomb-Like Films

**Figure 9:**
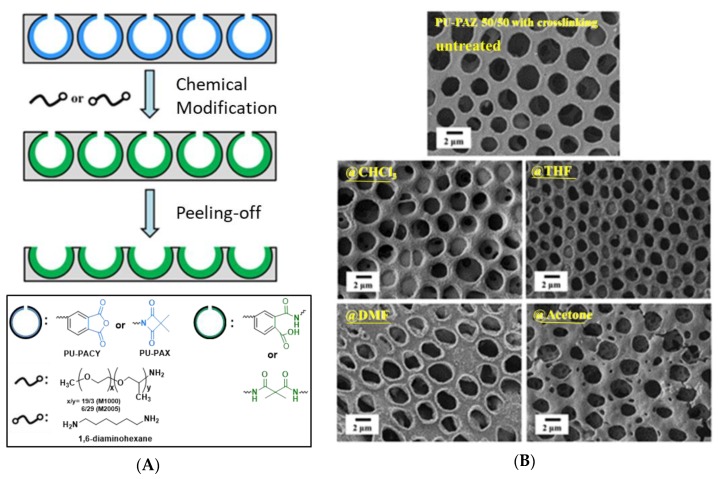
Schematic representations of crosslinked honeycomb-like films [26]: (**A**) Reactive pendent units of PUs; (**B**) images of crosslinked honeycomb-like films for solvent treatment. © Reproduced with permission from Elsevier.

Su et al. [26] developed two dendritic side-chain polyurethanes (PUs), poly(urethane-co-acylurea) (PU-PACY) and polyurethane-co-azetidine-2,4-dione (PU-PAZ), presenting reactive pendent units for fabricating wettability-tuning and solvent-resistant honeycomb-like films via the BF process (in chloroform). Through hydrophobic or hydrophilic modification of honeycomb-like films, the surface properties could be manipulated (Figure 9a). In addition, the solvent-resistant honeycomb-like films were obtained when PU-ACY or PU-PAZ films were treated with 1,6-diaminohexane for further crosslinking reaction. In Figure 9b, the crosslinked samples exhibited significant improvement in stability against the solvents. More recently, a crosslinked polyimide developed by Male et al. [62] also exhibited well retention of honeycomb-like morphology after 20 h immersion in organic solvents.

**Figure 10:**
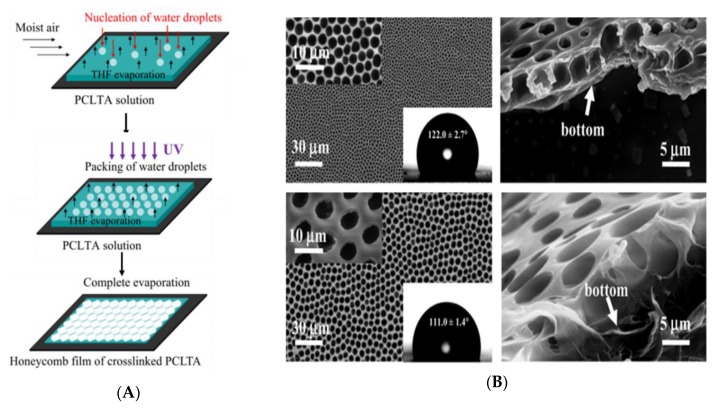
Schematic of photo-cured PCLTA honeycomb-like film [43]: (**A**) Processing steps; (**B**) images of honeycomb-like films. © Reproduced with permission from Elsevier.

An UV-curable poly(ε-caprolactone) triacrylate (PCLTA) was developed for regulating cellular behavior by Wu et al. [43]. Honeycomb-like films were fabricated from PCLTA solution via the BF method with photo-curing (Figure 10). It is worth noting that the volatile, water-miscible, relatively non-toxic solvent tetrahydrofuran (THF) was utilized in the study. The obtained biocompatible crosslinked PCLTA honeycomb-like films were also evaluated for mouse pre-osteoblastic MC3T3-E1 cell adhesion, spreading, proliferation, differentiation, and gene expression.

## 3. Applications

**Figure 11:**
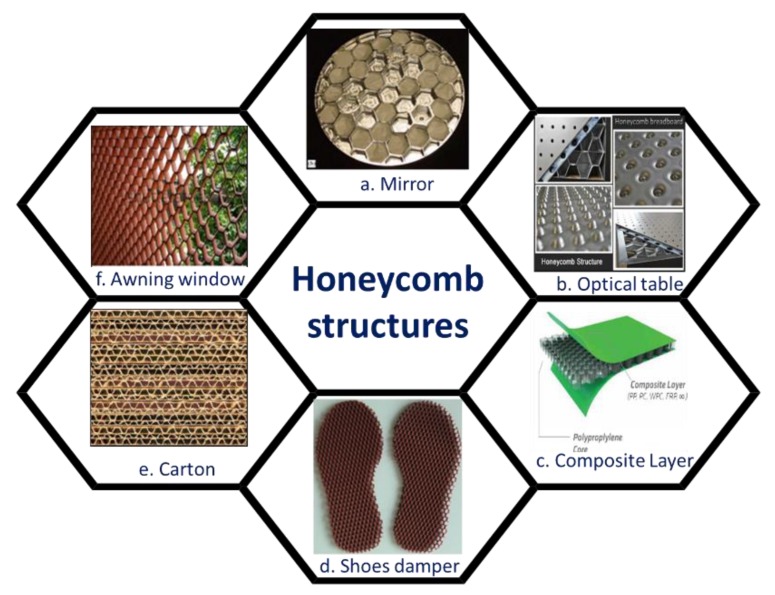
Honeycomb-like structures in our daily life.

The feature of the honeycomb structure is a two- or three-dimensional regular arrangement on material surfaces. In the honeycomb scale, a building can be realized from the architecture field with meter-scale to biomaterials with nanoscale [15]. Some commercial products with honeycomb-like structures are used in our daily life (Figure 11). For example, the honeycomb matrix was a useful structure for damping, the steel is always built in the form of honeycomb under the optical table (Figure 11b). PU foam with honeycomb structure also plays a role of cushioning in our shoes (Figure 11d). Furthermore, the design of the honeycomb structure with rectangular or hexagonal cells are useful for the enhancement of heat transfer, while the triangular honeycomb structure possesses better mechanical properties [15]. Apart from the above mentioned, the applications of polymeric honeycomb-like films would be a totally new frontier.

### Applications of Polymeric Ordered Arrays

**Figure 12:**
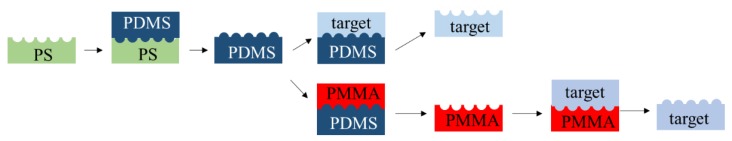
Honeycomb-like films as a template.

In recent years, polymeric honeycomb-like films via the BF process have received lots of scientific attention, especially in pursuit of practical applications [4,5,9,10,11,12,13,14,16,63]. These applications are classified in several fields, such as templating [64,65], surface-enhanced Raman scattering (SERS) [49,66,67,68,69,70], biomedical researches [43,71], electronic devices [72,73,74,75,76,77], etc. The ordered array films could serve as the templates to transfer certain patterns for polymers which are not easy to prepare directly by the BF method. For instance, a polydimethylsiloxane (PDMS) elastomer precursor was poured onto a PS honeycomb-like porous structures surface, and cured afterward (Figure 12). The PDMS microarrays were obtained and further transferred to other materials. This transferring process exhibits great potential for a wide variety of materials for replicating honeycomb-like films [78].

**Figure 13:**
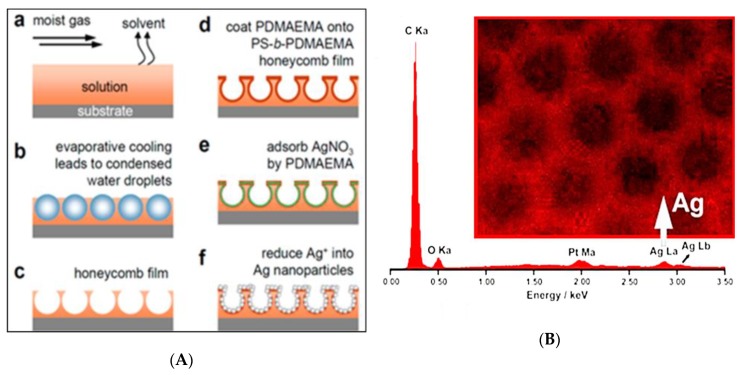
Schematic of the formation of honeycomb-like films with polymer-coated AgNPs [69]: (**A**) Formation of SERS substrates; (**B**) EDX mapping image of the Ag element on honeycomb-like film. © Reproduced with permission from the American Chemical Society (ACS).

Ou et al. [69] presented a SERS substrate which combined ordered structures and silver nanoparticles (AgNPs) generated in situ by the BF method (Figure 13). SERS substrates by combining hierarchically-patterned micro- and nanostructures with AgNPs adsorbed on a poly(*N*,*N*-dimethylaminoethyl methacrylate) surface would exhibit exciting surface-enhanced factors as high as 4 × 10^8^. This characteristic was shown for ordered array films including AgNPs, with diameters mostly ranging from 18 to 30 nm.

**Figure 14:**
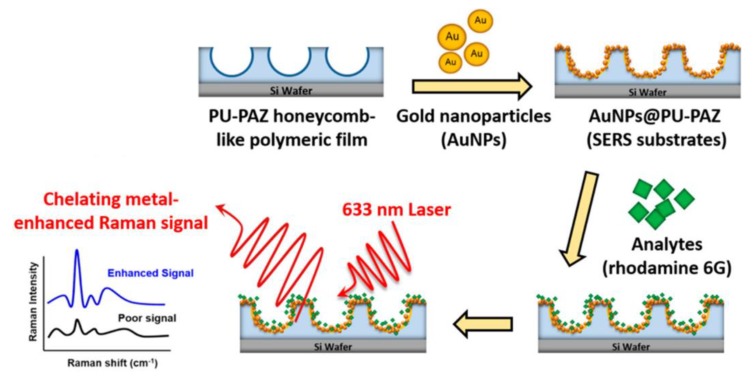
Diagram of the honeycomb-like surface-enhanced Raman scattering (SERS) substrate fabrication [49]. © reprinted from [49] under open access license.

Apart from that, an ordered arrangement of metal nanostructures would localize the surface plasmon resonance. This order array surface was utilized to investigate the signal of surface-enhanced Raman scattering (SERS). More recently, Chiang et al. [49] prepared substrates with a honeycomb-like surface for SERS detection by using amphiphilic dendron-containing polyurethane-co-azetidine-2,4-dione (PU-PAZ) (Figure 14). This study provided 3D nanoparticle arrays on honeycomb-like films for investigating the hot-spot effects by AuNPs. As a result, surface enhancement factors were greatly enhanced when compared with those of the flat-film substrates, due to the presence of the 3D porous structures.

**Figure 15:**
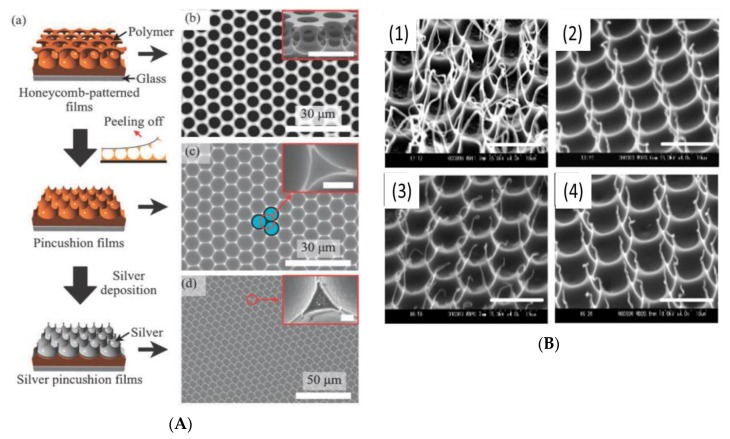
The SEM image peeling-off honeycomb-like patterned films [66]: (**A**) Silver pincushion films; (**B**) polymer pincushions of (1) PCL; (2) PLA; (3) PLGA and (4) PHB (bar: 10 mm) [71]. © Reproduced from [66] with permission from the Royal Society of Chemistry (RSC); © Reproduced from [66] with permission from the Wiley.

In addition, SERS substrates were also prepared by simply peeling off the top layer of the honeycomb-like films via Scotch tape [66]. Tanaka et al. [71] deposited silver on pincushion films and demonstrated the detection of rhodamine 6G (R6G) at concentrations as low as 0.5 nM (Figure 15a). For biomedical applications, these pincushions arrays could be made from biodegradable polymers such as poly(e-caprolactone) (PCL), poly(l-lactide) (PLA), poly(d,l -lactide-co-glycolide) (PLGA), and poly(3-hydroxy-butyrate) (PHB) (Figure 15b). These pincushion films could be used as cell-support scaffolds to produce nano- and micro-topographies. It is also important to note that a smart honeycomb-patterned surface can be achieved from PS-b-P4VP pH-responsive block copolymers using the breath figure process, along with pincushion arrays after the peeling-off process [79].

**Figure 16:**
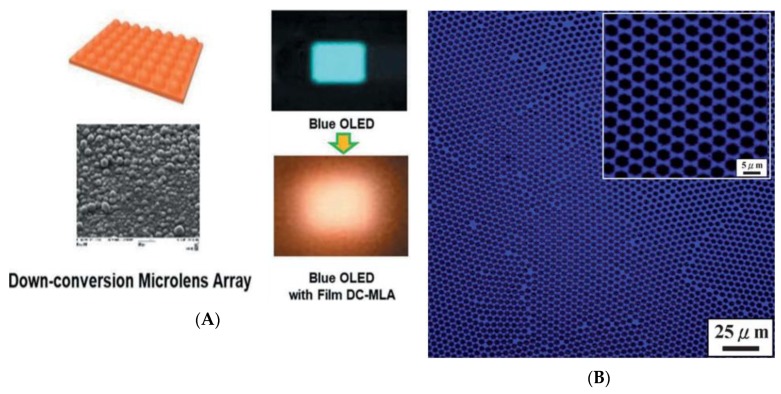
Honeycomb-like films for OLED: (**A**) Microlens array film for blue OLEDs and down-conversion white OLED [74]; (**B**) laser confocal fluorescence microscopy images of PF-b-PSA [77]. © Reproduced from [74] under open access license; © Reproduced from [77] with permission from the Royal Society of Chemistry (RSC).

On the other hand, the periodic microstructures were used in some optoelectronic devices, including microlens arrays (MLAs), and micropatterned light-emitting diodes (LEDs) [73,74,77,80]. MLA prepared by the BF method would act as the key component for the signal enhancement of optoelectronic devices such as optical telecommunication, displays, and solid-state lighting. The sizes of the periodic microstructures fell within the range of 100 nm to 10 μm, matching the requirements of optical and optoelectronic devices (Figure 16a). Therefore, the BF process provided a simple and inexpensive route to obtain ordered micropatterns for optical and optoelectronic devices. Apart from that, Chiu et al. [77] developed the rod-coil diblock copolymers of poly [2,7-(9,9-dihexylfluorene)]-block-poly(stearyl acrylate) (PF-b-PSA) to form highly-ordered microporous films (in chloroform) through the BF process. The blue-emission of the honeycomb-like film is shown in Figure 16b, and the emission band of these honeycomb-like films is dependent on the morphological properties, which can be tuned by the variation of the humidity and polymer concentration.

**Figure 17:**
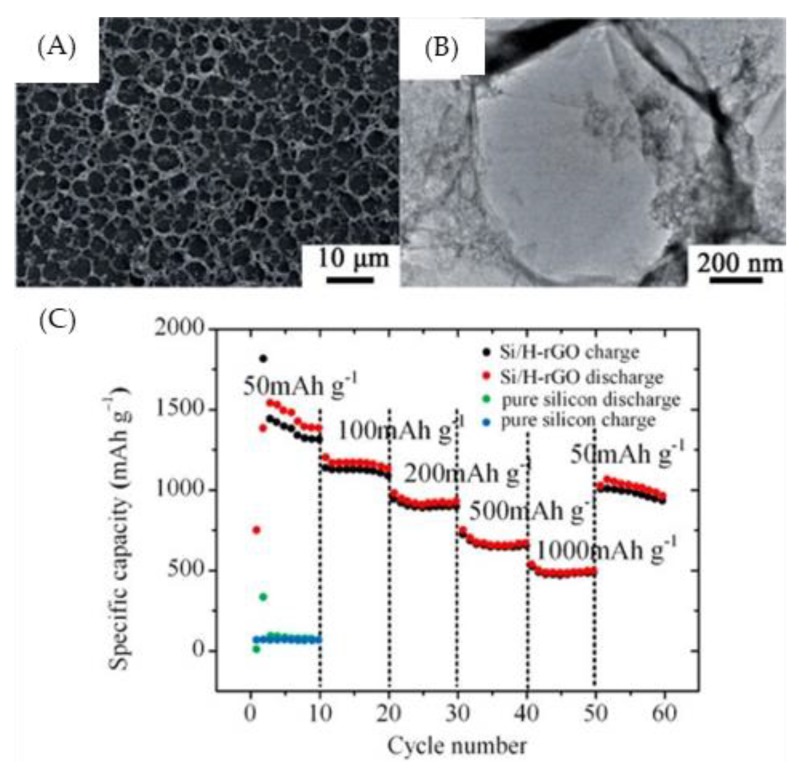
Images and performance of Si/H-rGO composite films. (**A**) SEM image of the Si/H-rGO composite; (**B**) TEM image of the Si/H-rGO composite; (**C**) rate capability of the Si/H-rGO composite film and pure silicon at various current densities ranging from 50 to 1000 mA g^−1^ [72]. © Reproduced with permission from the Royal Society of Chemistry (RSC).

Honeycomb-like films via the BF process also played an important role as silicon-based anode materials or solid-state electrolytes for the booming development of lithium ion batteries (LIBs) [72,75,81,82,83,84,85,86]. A silicon–honeycomb graphene composite film was developed as a high-performance anode material for lithium ion batteries [72]. The honeycomb graphene structure is capable of circumventing the agglomeration of the silicon nanoparticles, enhancing the electrical conductivity and decreasing the transfer resistance of Li^+^. Consequently, the well-mixed Si/GO/surfactant honeycomb-like composite film presented a high specific capacity and good cycling stability for lithium ion batteries (Figure 17).

**Figure 18:**
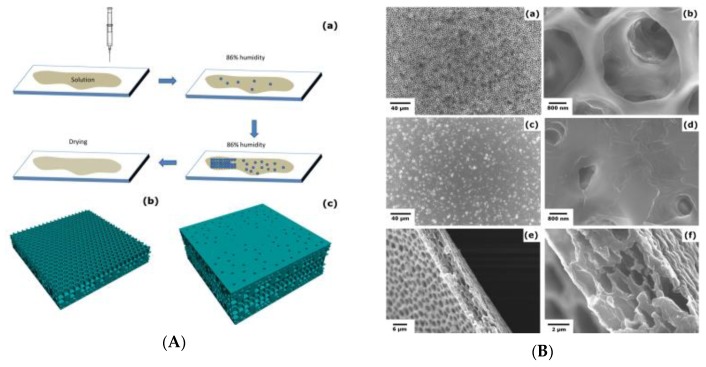
The preparation process of the PVDF-HFP porous polymer membrane [75]: (**A**) Schematic illustration; (**B**) FESEM images of (a and b) the front side, (c and d) the back side, and (e and f) the cross-section. © reprinted from [75] under open access license.

Zhang et al. [75] reported on poly(vinylidene difluoride-co-hexafluoropropylene) (PVDF-HFP) polymer membranes with multi-sized honeycomb-like architectures (Figure 18). These polymer electrolyte membranes possessed a porosity of 78%, leading to high electrolyte uptake (86.2 wt%). As a gel polymer electrolyte, this honeycomb-like PVDF-HFP membrane exhibited a high ionic conductivity of 1.03 mS/cm at room temperature, which was much higher than that of commercial polymer membranes (<0.1 mS/cm). Most importantly, the highlight in this study was the usage of relatively benign acetone as the solvent. In addition to acetone, less hazardous solvents such as ethyl acetate (EA) and THF were also chosen to prepare honeycomb-like porous polymer electrolyte membranes by the BF method [81,83,84,86].

**Figure 19:**
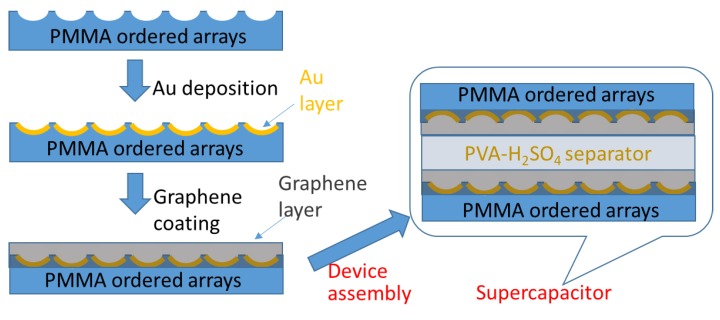
PMMA honeycomb-like films as an electrode for a supercapacitor.

Abbaspour et al. [76] developed honeycomb-like surfaces on transparent poly(methyl methacrylate) (PMMA) films using a facile direct breath figure (DBF) method. This was utilized as an electrode for solid-state supercapacitors (Figure 19). The pore size of the ordered arrays on the PMMA surface exhibited diameters in a range of 0.5 to 10 μm. Subsequently, a graphene layer was deposited on the surface by spray-coating. The solid-state supercapacitor with a honeycomb-like surface showed superior specific capacitance when compared with the flat one.

**Figure 20:**
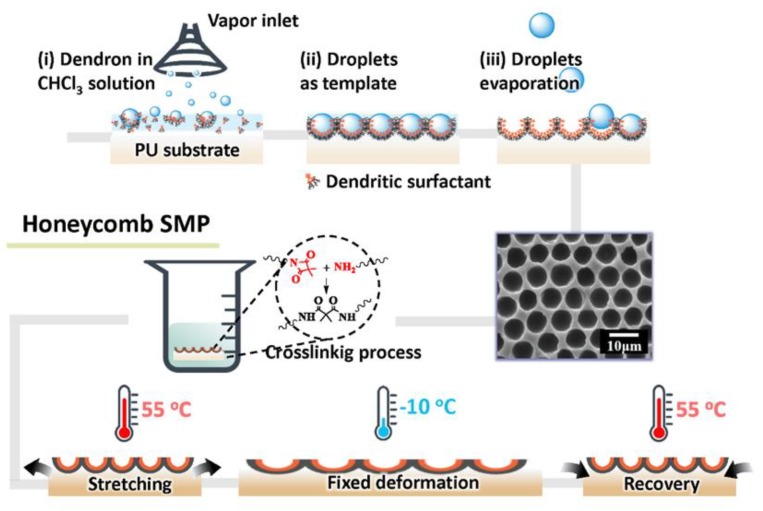
Preparation of honeycomb-like surface with shape memory behavior [29]. © Reproduced with permission from Wiley.

More recently, Wu et al. [29] also adopted a modified DBF process to prepare a honeycomb-like structure on a PU substrate with shape memory effect (Figure 20). This approach is set forth to deal with the thickness issue for the traditional BF method. A chemical cross-linkable shape-memory PU with active side chains was utilized as the substrate material. A small amount of an amphiphilic dendron was utilized as surfactant to form a honeycomb-like structure on the PU substrate via a BF process [28]. As a result, a honeycomb-like structure with shape memory behavior and switchable wettability was realized.

## 4. Towards a Greener Process

### 4.1. Investigation of Water Droplet Nucleation

**Figure 21:**
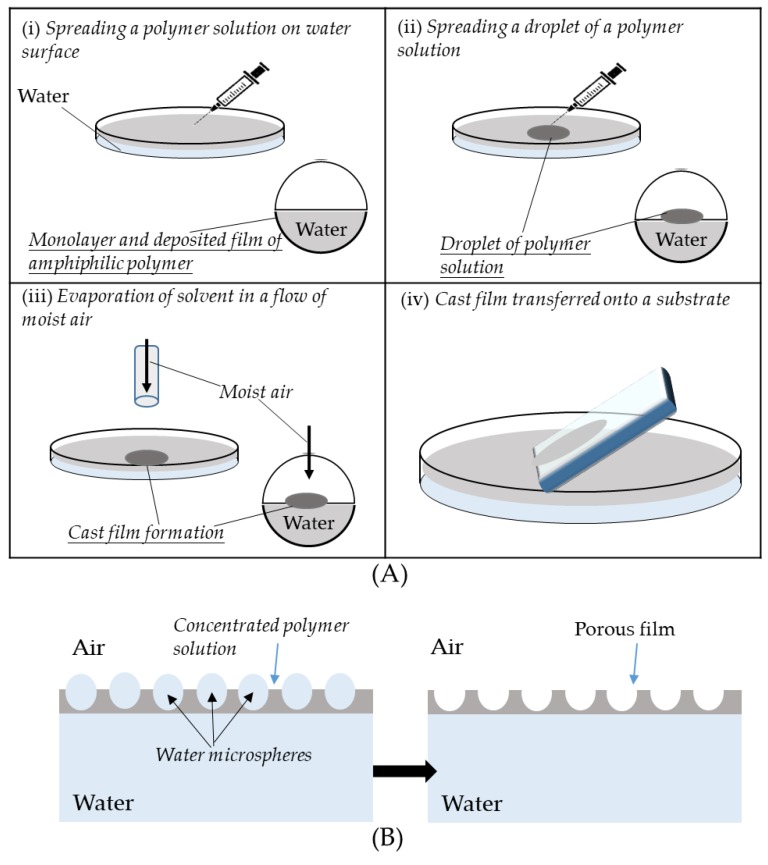
Honeycomb-like film was fabricated with water as the template [88]: (**A**) Fabrication process of a microporous film on a water surface; (**B**) formation of the porous morphology of a polymer film. © Reproduced with permission from the American Chemical Society (ACS).

As mentioned previously, honeycomb-like film made from the BF process relies on the interfacial stability between water droplets and solutions. In addition to the consideration of polymer design, substrates and solvents are two critical factors that influence the morphologies of honeycomb-like films [14]. Ferrari et al. [87] reported the use of various substrates with different surface energies. In general, substrates typically exhibit a qualitative effect (nature, hydrophilicity, wettability) on the formation of honeycomb-like films. Different morphologies of honeycomb-like films could be derived from the substrates with various reagent treatments, such as glasses washed with piranha solution, silicon wafer washed with H_2_O_2_-NH_4_OH-H_2_O solution (RCA1), glasses silanized with alkoxysilanes, or glasses functionalized with fluorinated silanes. Furthermore, some flexible substrates based on polyethylene (PE), polyvinylchloride (PVC), or polyethylene terephthalate (PET) are also utilized for the formation of desirable honeycomb-like structures. Apart from that, honeycomb-like films are realized on a liquid substrate (air–water interface) by Nishikawa et al. [88]. A self-standing honeycomb-like film was formed via the so-called “on-water spreading” method (Figure 21).

**Figure 22:**
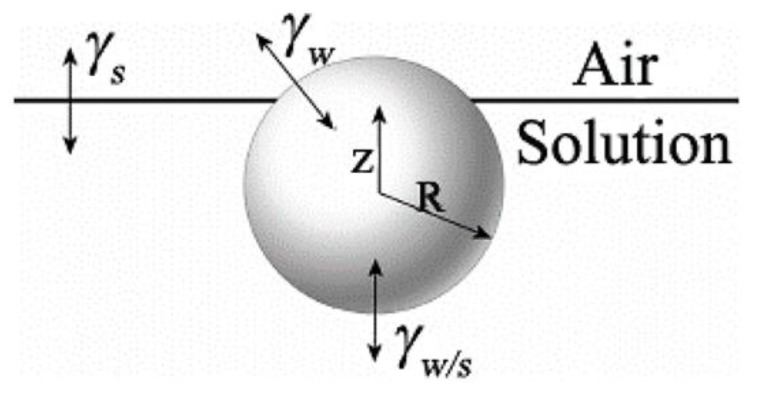
The relationship between a water droplet and polymer solution surface [89]. © Reproduced with permission from the American Chemical Society (ACS).

CS_2_ and chloroform were the most commonly-used solvents in preparing honeycomb-like films due to their water immiscibility, lower boiling point, and good solubility for polymers. The first polymeric honeycomb-like films were developed by exposing a drop of polystyrene-b-polyparaphenylene solution in CS_2_ to a flow of moist air by Francois et al. in 1994 [3]. The pore size and shape depend on the self-assembly process between water droplets–solvent interface [89,90]. During the nucleation condensation process, the pore regularity and size were dominated by the interfacial properties between water droplets and polymer solutions. During this stage, the solvent played a critical role in maintaining the interfacial balance between solvent–water and solvent–substrate for a polymer solution. In particular, the interfacial properties between the water droplets and the solvent would mainly influence the morphology of honeycomb-like films. The interfacial energy balance (z_0_) can be defined as: z_0_ = z/R = (*γ*_w_ − *γ*_w/s_)/*γ*_s_, where z is the distance between the droplet center and the air–solution interface; R is the droplet radius; *γ*_w/s_ is the interfacial tension between water and solution; *γ*_w_ and *γ*_s_ are the surface tension of the water and the solution, respectively (Figure 22). Consequently, the shape of ordered arrays can be predicted by the calculation of interfacial energy balance.

**Figure 23:**
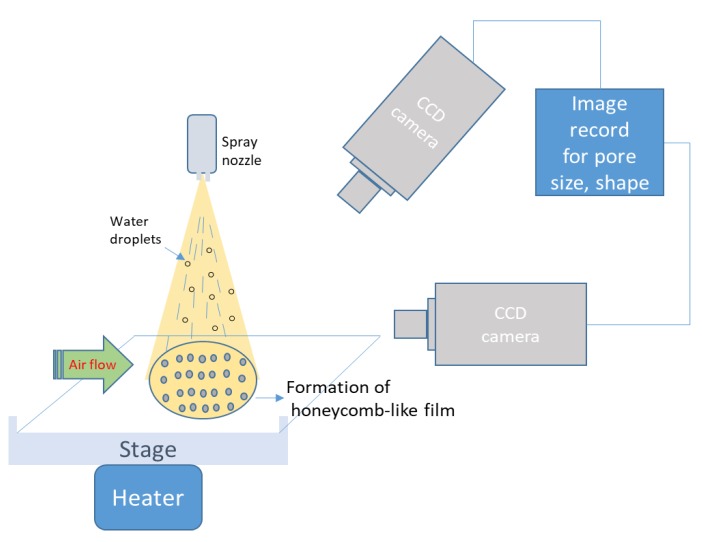
Schematic of the CCD camera system for real-time image during the BF process.

In order to understand the nucleation mechanism of water droplets on the solution surface and evaporation during the BF process, a high-speed camera system was set up to observe the real-time images [91,92,93,94,95]. Therefore, the formation of ordered array pores could be closely monitored. The layout of a high speed camera with temperature control equipment and the actual system setup are shown in Figure 23.

### 4.2. The Concept of Green Solvents

The solvent properties of boiling point, density, miscibility with water, and the thermodynamic affinity with a polymer solution have to be taken into account when it comes to the choice of solvent for proper water–solution interfacial tension. Because of this, most of the reported honeycomb-like films via the BF process were obtained using volatile solvents such as CS_2_, chloroform, dichloromethane, benzene, and chlorobenzene [87,96,97,98,99]. However, according to various solvent selection guides, including the CHEM21 selection guide of classical and less classical solvents, GlaxoSmithKline (GSK), AstraZeneca, and the American Chemical Society Green Chemistry Institute (ACS GCI), these commonly-used solvents were considered as highly hazardous (marked in bold in Table 1) [100,101,102,103].

### 4.3. Greener Solvents for the BF Process

#### 4.3.1. Use of Less Hazardous Solvents (Toluene, THF, Acetone, Acetonitrile, EA, and MEK)

**Figure 24:**
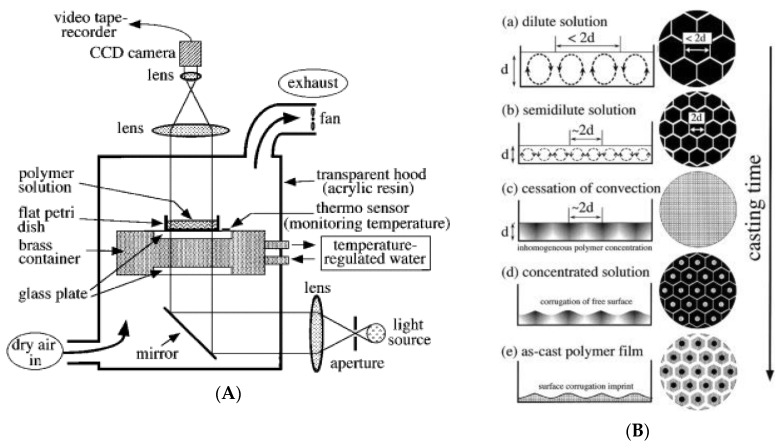
Toluene for honeycomb-like films: (**A**) Experimental set up for in situ observation of the surface corrugation pattern process; (**B**) schematic illustrations of casting solutions and results [106]. © Reproduced with permission from Elsevier.

Based on the solvent selection guide above, several researches revealed the possibility of using less hazardous solvents such as toluene [87,104,105,106,107,108], and acetone [75,81,86,87,109], THF [43,63,84,87,105,107,108,110,111,112,113,114,115], acetonitrile [116], EA [83,87,109,114,117], and MEK [87] for the BF process (marked in bold in Table 1). Sakurai et al. [106] found that by using toluene as the solvent, the mesh size was increased during the lower evaporation rate of kinetic control. Meanwhile, the pore size decreased tremendously with increasing rate under the higher evaporation rate due to temperature-gradient control (Figure 24).

**Figure 25:**
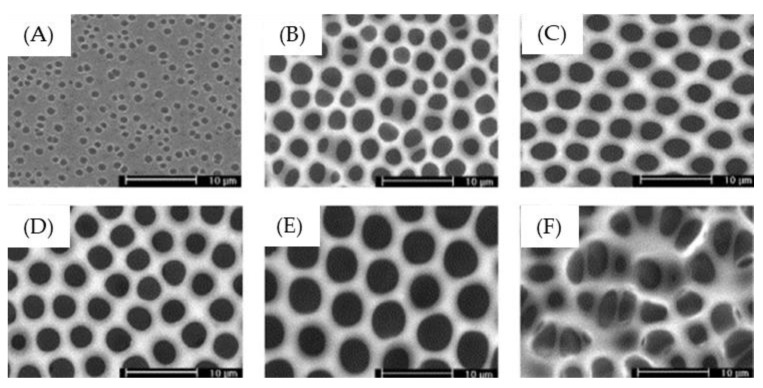
The SEM images of SAN copolymer in THF solution (0.08 g/ml) under various relative humidity at 25 °C after BF processing: (**A**) 30%; (**B**) 40%; (**C**) 50%; (**D**) 60%; (**E**) 70%; (**F**) 80% [118]. © Reproduced with permission from Elsevier.

THF, a water-miscible solvent, has been widely utilized for polymer processing because it is a moderately polar solvent and can dissolve a wide range of nonpolar and polar polymers. Zhao et al. [118] prepared a random copolymer poly(styrene-*co*-acrylonitrile) (SAN) in THF for the fabrication of honeycomb-like films via the BF process (Figure 25). Well-defined macroporous membranes with good mechanical properties were achieved under 50–70% relative humidity.

**Figure 26:**
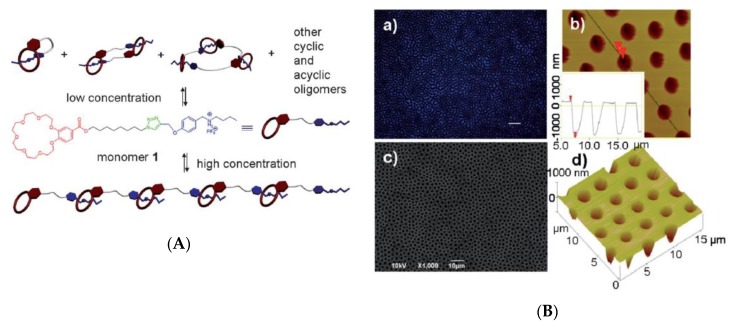
Supramolecular polymer for honeycomb-like films [116]: (**A**) Self-organization of supramolecular; (**B**) images of honeycomb-patterned films. © Reproduced with permission from the Royal Society of Chemistry (RSC).

Acetonitrile is considered less hazardous when compared with benzene, chloroform, CS2, and dichloromethane. Honeycomb-like films were fabricated from a highly-ordered supramolecular polymer soluble in acetonitrile via the BF process [116]. In fact, this is a premier study of supramolecular polymer honeycomb-like films via the BF process. The integration of the reversible formation and stimuli-responsiveness of supramolecular polymers with ordered arrays is of great potential in applications (Figure 26).

**Figure 27:**
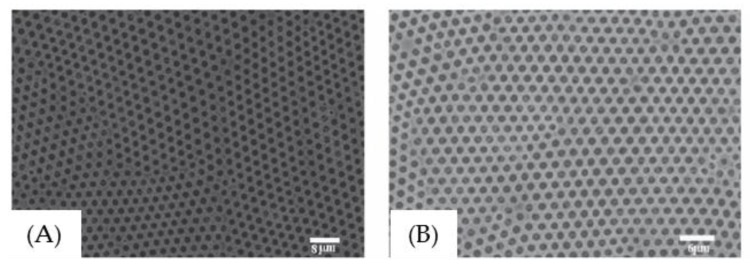
TEM images of PVPh-b-PS honeycomb-like films [105]: (**A**) 20 mg in THF + 0.1 mL toluene; (**B**) 50 mg in THF + 0.1 mL toluene. © Reproduced with permission from Elsevier.

The utilization of mixed solvents in polymer processing is often made for morphological control [119,120,121]. This is because the mixed-solvent system comprises two solvents of different boiling points and polarity. In some cases, the researchers would rather opt for a less hazardous solvent to be involved. A mixed solvent system of THF and toluene was utilized for fabricating polymeric ordered arrays via the BF process by Tung et al. [105]. The amphiphilic diblock copolymer poly(vinyl phenol)-block-polystyrene (PVPh-b-PS) was first dissolved in THF. Subsequently, a small amount of toluene was added into the stirring polymer solution to incur partial precipitation of the PVPh blocks (i.e., formation of micelles). A three-dimensional honeycomb-like morphology was achieved via the BF process (Figure 27). The concept of the mixed-solvent system for fabricating honeycomb-like films via the BF process provides the possibility of using a greener solvent for polymeric ordered arrays.

**Figure 28:**
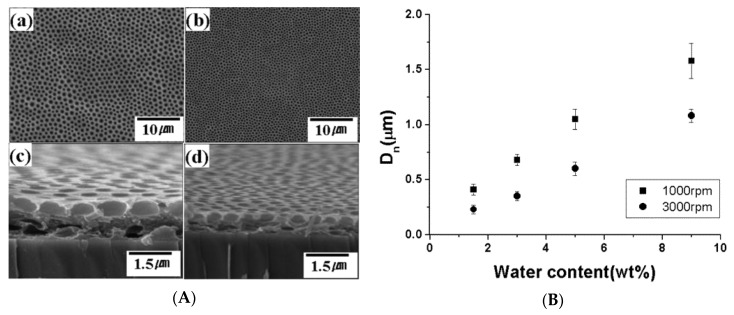
Honeycomb-like films by spin coating: (**A**) SEM images of the surface and cross section of cellulose acetate butyrate-based films (a,c): 1000 rpm, (b,d): 3000 rpm; (**B**) plots of the number average diameter (D_n_) versus water content with various rotating speeds for honeycomb-like films fabricated under a dry condition (RH = 30%) [109]. © Reproduced with permission from the American Chemical Society (ACS).

Another ingenious approach for a robust mixed-solvent system was reported by Park et al. [109]. Honeycomb-like films by the BF process were prepared by spin-coating of polymer solutions (THF or acetone) under a dry condition with a relative humidity (RH) of 30% (Figure 28). It is important to note that small amounts of water were added to water-miscible solvents such as THF or acetone. The water content, relative humidity, and the spinning rate were closely related to the pore sizes after the drying of solvent. Thus, this unique approach exhibited great potential for fabricating large-scale honeycomb-like films with various pore sizes. In addition, Madej et al. [121] also reported the morphology control of PMMA blend ordered arrays (in THF), by not only mixing with a certain amount of water content (3 wt% ≤ H_2_O ≤ 20 wt%), but changing the relative humidity in the range of 5% to 80% as well.

#### 4.3.2. Stabilizing Water Droplets with Surfactants and Colloidal Particles

**Figure 29:**
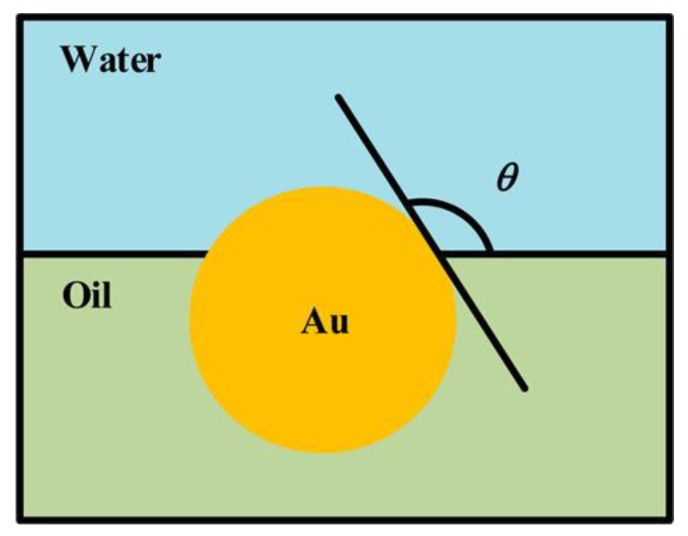
A small gold particle at the water–oil interface [125]. © Reproduced with permission from the American Chemical Society (ACS).

Fukuhira et al. [122] investigated the manipulation of interfacial tension between water and polymer solution (in toluene) for the preparation of honeycomb-like films. By the addition of a small amount of phospholipid surfactants to poly(d,l-lactic acid) (PLA), the dierucoylphosphatidylethanolamine- and dioleoylphosphatidylethanolamine-containing PLA solutions all exhibited high interfacial tension in the fabrication of biocompatible honeycomb-like films. On the other hand, the usage of nanoparticles would able to stabilize the interface between solution and water droplets without changing the interfacial tension [40,123,124]. The presence of colloidal particles provides more stabilization energy (i.e., the Pickering-emulsion effect) during the formation of water condensation on the solution surface. Once the spherical colloidal particle adsorbs in the interface, the energy required to remove the particle from the interface is given by Equation (1):

(1) $$E=\pi R^{2}\gamma_{WO}{\left(1-\left|cos\theta \right|\right)}^{2},$$

where *R* refers to the radius of particle, $\gamma_{WO}$ refers to the interfacial tension between water and solution, θ is the contact angle through the water phase (Figure 29).

A similar colloidal particles approach was also attempted by Li et al. [126]. Honeycomb-like films were fabricated by using dodecanethiol-capped gold nanoparticles in toluene. The pore morphology with circle or ellipse shapes would be dependent on the direction and velocity of vapor.

**Figure 30:**
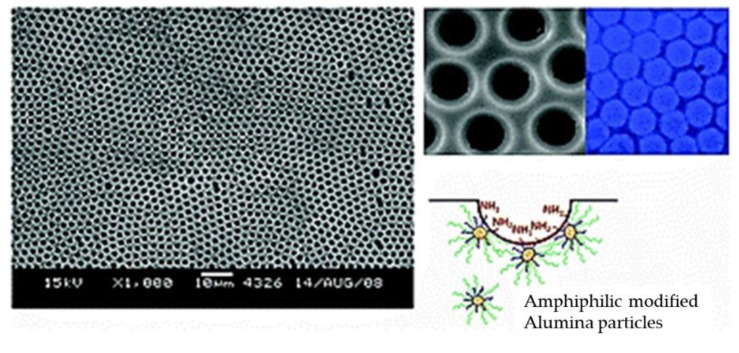
SEM images of the honeycomb-like films fabricated from the PS–alumina hybrid [110]. © Reproduced with permission from the Royal Society of Chemistry (RSC).

In another example, Lakshmi et al. [110] fabricated polystyrene–alumina nanocomposite films with ordered arrays, which were prepared from suspensions of amphiphilic-modified alumina particles in polystyrene solutions via the so-called particle-assisted BF process. The key factors for influencing morphological phenomena are particle concentration and the hydrophobic–hydrophilic balance of the amphiphilic-modified alumina particles in the polar or nonpolar solvents (Figure 30).

### 4.4. Unique Techniques for Ordered Arrays

**Figure 31:**
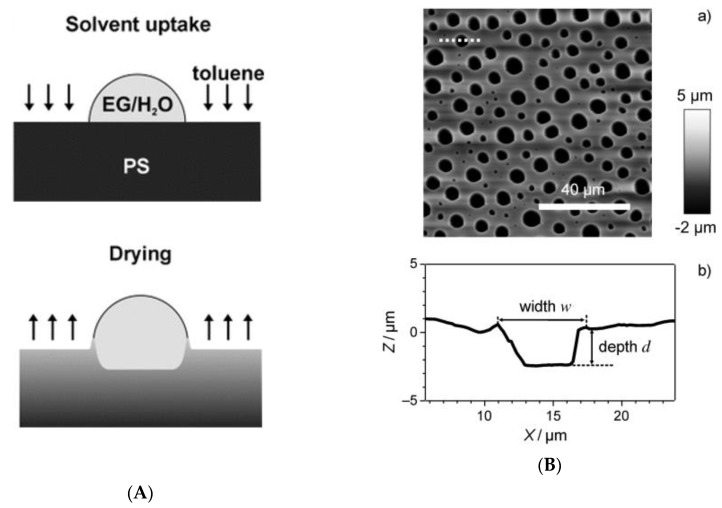
(**A**) Illustration of the toluene (arrows) into a polystyrene (PS) substrate, covered with a sensible drop of an equal amount mixture of EG and water (EG/H_2_O), and drying of the expanded polymer surface; (**B**) atomic force microscopy (AFM) image of a dried PS surface after a 5 min exposure to toluene vapor and consecutive condensation of water microdroplets, and its height profile of a pore along the dashed white line in [127]. © Reproduced with permission from Wiley.

Other than the typical BF process, there are some unique routes to achieve polymeric ordered arrays. Pericet-Camara et al. [127] investigated that toluene-vapor-softened polystyrene surfaces were micropatterned with nonsolvent sessile droplets. Through sequentially depositing non-evaporating droplets of EG/H_2_O on the original polystyrene surfaces, and exposing the surfaces to saturated toluene vapor, ordered arrays could be obtained (Figure 31).

In another example, Castaño et al. [128] reported a clean methodology by the combination of supercritical CO_2_ (SCCO_2_) foaming technology and the BF process. The formation of inner porosity for poly(ε-caprolactone) (PCL) was obtained via the SCCO_2_ technique, whereas the outer porosity was produced via the BF process. Despite that the dipping process in the chloroform was required for the BF process, this porous PCL material provided spaces for tissue penetrating in the scaffold, and improved cell adhesion and proliferation until its degradation.

## 5. Summary and Future Prospects

The BF method for fabricating polymeric honeycomb-like films has been drawing great attention since Francois initially created ordered hexagonal honeycomb-like films in 1994. Even though an uncertainty in precise surface morphology control is present for the polymeric ordered arrays, the BF process is a reliable method because of its low cost and certain degree of maneuverability. With great advances in the BF process, several practical applications of honeycomb-like films via the BF method were realized, such as templating, surface-enhanced Raman scattering (SERS), biomedical researches, and electronic devices. In this review, we attempted to search for greener BF processes along with less hazardous solvents for the sake of safety, health, and the environment. The utilization of less hazardous solvents such as toluene, THF, acetone, acetonitrile, EA, and MEK for fabricating honeycomb-like films via the BF process indicates that there are alternatives for the commonly-used “highly hazardous” solvents such as chloroform, CS_2_, and benzene. Moreover, certain greener solvents can be candidates for the BF process based on the concept of the mixed-solvent system, which comprises two solvents of different boiling points and polarity. Apart from that, greener BF processes can be facilitated by the addition of small amounts of surfactants or colloid particles to the polymer solutions, especially with the right choice of certain green solvents. Based on the above, we strongly believe that green BF processes will be within reach soon enough.

## Figures and Tables

**Table 1:** Solvent category from the CHEM21 selection guide [100].

Category	Solvents
Recommended	Water, alcohols (EtOH, i-PrOH, n-BuOH), **ethyl acetate (EtOAc**; **EA)**, isopropyl acetate (i-PrOAc), butyl acetate (n-BuOAc), anisole, sulfolane.
Recommended or problematic?	MeOH, tert-butyl alcohol (t-BuOH), benzyl alcohol, ethylene glycol (EG), **acetone**, **methyl ethyl ketone (MEK)**, methyl isobutyl ketone (MIBK), cyclohexanone, methyl acetate (MeOAc), acetic acid (AcOH), acetic anhydride (Ac_2_O).
Problematic	2Me-THF, heptane, Me-cyclohexane, **toluene**, xylenes, chlorobenzene, **acetonitrile**, N,N′-dimethylpropyleneurea (DMPU), dimethyl sulfoxide (DMSO).
Problematic or hazardous?	Methyl tert-butyl ether (MTBE), **THF**, cyclohexane, dichloromethane (DCM), formic acid, pyridine
Hazardous	Diisopropyl ether, 1,4-dioxane, dimethyl ether (DME), pentane, hexane, *N*,*N′*-dimethylformamide (DMF), *N*,*N′*-dimethylacetamide (DMAc), *N*-methyl-2-pyrrolidone (NMP), methoxy-ethanol, triethylamine (TEA).
Highly hazardous	Diethyl ether, **benzene**, **chloroform**, tetrachloromethane (CCl_4_), 1,2-dichloroethane (DCE), nitromethane, **carbon disulfide (CS_2_)**, hexamethylphosphoramide (HMPA)
